# Elevated IOP Alters the Material Properties of Sclera and Lamina Cribrosa in Monkeys

**DOI:** 10.1155/2022/5038847

**Published:** 2022-08-23

**Authors:** Xu Jia, Feng Zhang, Mengdan Cao, Zheng Pan, Ke Liu, Dengming Zhou, Xuanchu Duan

**Affiliations:** ^1^Department of Ophthalmology, The Affiliated Hospital of Guizhou Medical University, Guiyang, 550000 Guizhou, China; ^2^Department of Ophthalmology, The Third Xiangya Hospital, Central South University, Changsha, 410000 Hunan, China; ^3^Department of Ophthalmology, The Second Xiangya Hospital, Central South University, Changsha, 410000 Hunan, China; ^4^Aier School of Ophthalmology, Central South University, Changsha, Hunan, China; ^5^Changsha Aier Eye Hospital, Glaucoma Research Institute, Aier Eye Hospital Group, Changsha, 410000 Hunan, China

## Abstract

**Objective:**

Elevated intraocular pressure (IOP) has significant impacts on different stages in the progression of chronic glaucoma. In this study, we investigated changes in the material properties of sclera and lamina cribrosa (LC) in a nonhuman primate model with elevated IOP.

**Methods:**

Normal adult Tibetan macaques were selected for the construction of elevated IOP model. After 40 days of stable maintenance on the ocular hypertension, the binocular eyeballs were obtained for the measurement of macroscopic parameters of the eyeballs. Posterior scleral tissue strips were obtained in circumferential and axial directions, and thickness was measured, respectively. Biomechanical parameters were obtained with stress relaxation, creep, and tensile test. The nanoindentation test was performed on the LC and scleral tissue around optic nerve head (ONH) to obtain compressive modulus.

**Results:**

In the presence of elevated IOP, variations of the axial diameter of the eyeball were greater than those of the transverse diameter, and the mean scleral thickness around ONH was smaller in the experimental group than control group. The elastic modulus and stress relaxation modulus of sclera were larger, and the creep rate was lower in the experimental group than control group. In the control group, the elastic modulus and stress relaxation modulus of the circumferential sclera were larger in the axial direction, and creep rate was smaller. In the experimental group, there was no significant difference in biomechanical characteristics between the two directions. Compared to the control group, the compression modulus of the LC was smaller, and the compression modulus of sclera around ONH was larger in the experimental group.

**Conclusion:**

Elevated IOP alters the viscoelasticity and anisotropy of sclera and LC. These may contribute to reduction of the organizational resistance to external forces and decline in the ability of self-recovery.

## 1. Introduction

Glaucoma was a complex neurodegenerative disease characterized by of retinal ganglion cell (RGC) apoptosis and optic nerve damage. It was estimated that approximately 111.8 million population would suffer from glaucoma by 2040 around the world [1]. Studies have confirmed that the main cause of glaucoma was mechanical compression injury to lamina cribrosa (LC) by pathological elevated intraocular pressure (IOP) [2]. With the IOP increased, the LC, located in the posterior eyeball, was subjected to outward pressure, resulting in backward bending and expansion of LC. Thus, the biomechanical factors have emerged as a key aspect in the glaucomatous pathogenesis. Deformation of LC can cause edema of optic nerve fibers passing through it and impair local blood circulation, leading to optic nerve lesion [3]. Posterior sclera, which formed LC, was critical of LC structure and optic nerve protection. Sclera, composed of dense bundles of collagen fibers, plays an important role in maintaining the structural, functional, and biomechanical properties of the eyeball. And these fibers were crossing over each other and the amount varies among regions, making the sclera nonlinear, anisotropic, and viscoelastic. Current researches on biomechanical properties of sclera and LC in glaucoma were mostly based on the premise of unchanged tissue characteristics, ignoring the fact that tissue characteristics may change undergoing chronic course of disease [4, 5]. Clinically, it has been found that the same IOP reduction in the various stage of glaucoma shows different therapeutic effects [6], suggesting that the biomechanical characteristics of the sclera and LC vary in patients at different stages. However, variation trends of biomechanical characteristics were currently unclear with histological change of sclera and LC. In this study, Tibetan macaques, highly homologous to humans, were used to establish the ocular hypertension model, simulating the condition of human glaucoma disease with elevated IOP. Exploring the effect of elevated IOP on diameter of the eyeball and thickness of peripapillary sclera (ppSCl). Tensile tests and the latest bionanoindentation test were adopted to get the mechanical properties of ppSCl and LC in different quadrant in this research and comparing these parameters between animals in elevated IOP group and in normal IOP group. Collectively, this study will provide a better understanding about effects of chronic increased IOP on ppSCl and LC biomechanical characteristics in human glaucoma.

## 2. Methods

### 2.1. Construction of Elevated IOP Model in Animal

This study is approved by the Animal Care and Treatment Committee of Guizhou Medical University (no. 1800132). All experiments were performed in accordance with laboratory animal Care guidelines to minimize animal stress and distress. In this experiment, 8 of adult Tibetan macaques (16 eyes) were selected as experimental objects (provided by Sichuan Animal Experiment Center, China). 3 were 9 years old, 2 were 7 years old, and 3 were 8 years old, with an average age of 8.13 ± 0.83, and an average body weight of 8.32 ± 1.45 kg. Each monkey is performed with routine physical examination, excluded diabetes, hypertension, and other systemic diseases. They have gentle temperament, normal diet, and normal binocular vision function, and the fluctuation of bilateral IOP is in the range of 10-21 mmHg (measured by iCare rebound tonometor, Finland), excluding abnormal signs of conjunctiva hyperemia, corneal opacification, and abnormal pupillary light reflex. The right eye is included in the experimental group to establish elevated IOP model, and the left eye is included in the matched self-control group with no treatment. After general anesthesia with Zolazepam/Tiletamine (Zoletil 50, Virbac, France. Induction dose: 10-15 mg/kg, maintenance dose: 4-6 mg/kg), 0.2 ml aqueous humor is extracted from the anterior chamber in the right eye, and 0.2 ml *α*-chymotrypsin injection (Tiancheng Pharmaceutical Co. Ltd., China) containing 150 units is injected into the anterior chamber of the right eye (based on previous literature reports and our experimental experience [7–9]), and no treatment is done on the left eye. Bilateral IOP is measured 1 day, 3 days, 7 days, 14 days, 21 days, 28 days, 35 days, 42 days, 49 days, and 56 days after operation. The anterior chamber is observed with slit lamp (Topcon, Japan), and the IOP is required to be maintained in the range of 35-40 mmHg more than 40 days. If the IOP is too high or too low, the IOP can be maintained by paracentesis of anterior chamber or *α*-chymotrypsin injection through anterior chamber again.

### 2.2. Acquisition and Treatment of the Eyeballs

The animals were euthanized by overdose of pentobarbital sodium (50 mg/kg, Yaxin Pharmaceutical Co. Ltd., China) injection via intravenous after 40 days with stable maintenance of high IOP. Their eyeballs were removed, and muscles and conjunctiva fascia attached the eyeball were carefully removed under microscope to expose the sclera completely. The diameters of the eyeballs in axial and equatorial direction were measured with digital vernier caliper. The sclera is divided into four quadrants in accordance with the inferior, superior, nasal, and temporal sides and marked. In aseptic and HBS buffer (SAB, USA) environment, cut the scleral margin along the corners of the eyeball with the microsurgical scissor; the cornea and intraocular tissues were removed carefully.

**Figure 1:**
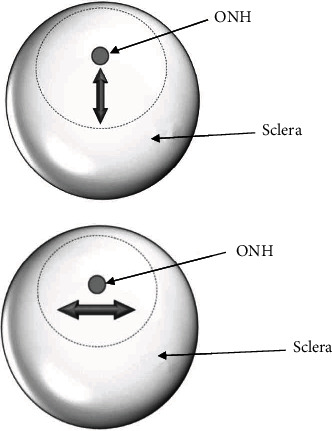
Schematic diagram of sclera strips preparation, the double-headed arrows represent the direction of scleral strips acquisition. (a) Peripapillary sclera strip in axial direction. (b) Peripapillary sclera strip in circumferential direction.

**Figure 2:**
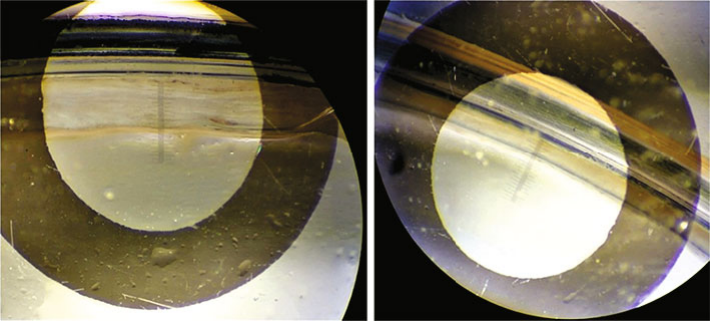
Scleral thickness measurement. Sclera strips were annularly attached on the interior wall of sterile petri dish, and the thickness values were measured with micrometer scale under stereomicroscope.

In order to explore differently mechanical characteristics of ppSCl tissue in axial (anteroposterior) and circumferential direction, respectively, the eyeballs in the experimental group and the control group were subdivided into two groups separately. Experimental group 1 and control group 1 (2 pairs of the eyeballs each): based on the four marked quadrants and centered on the ONH, ppSCls were cut radially into four directional tissue blocks. Each scleral block is cut into 10 mm long and 4 mm wide scleral strips with ophthalmic scissors (Figure 1(a)). Experimental group 2 and control group 2 (2 pairs of the eyeballs each): based on the four marked quadrants and centered on the ONH, ppSCls were cut into 4 tissue strips (10 mm long and 4 mm wide) in circumferential direction at 2 mm away from ONH with ophthalmic scissors (Figure 1(b)). The remaining ONH tissues and scleral tissues were put in tissue storage solution (Absin, China) temporarily for further experiment. Each sclera strip is attached on the interior wall of sterile petri dish, and the thickness values were measured with micrometer scale under stereomicroscope (Figure 2). 5 loci of each strip were selected for thickness measurement, and the mean value is taken by 3 times of measurement per locus.

### 2.3. Stress Relaxation Test and Tensile Test

In order to obtain viscoelastic properties of scleral strips, stress relaxation properties need to be tested. In this part of the experiment, tissue electronic mechanical testing systems (MTS Insight 30, USA) is used as the experimental tool. Both ends of scleral strips were fixed in self-made tensile test extension clips, respectively (about 1.5 mm), allowing the model to conform to standard of mechanics experiment (GBT 528-1998). Digital vernier caliper (accurate to 0.01 mm) is used to measure the width of each strip and the effective length between the clips. The mean value is taken by 3 times of measurement per strip, and then, the strips were placed into the HBS buffer solution for the next experiment. The upper and lower fixtures of MTS held the extension clips of scleral strips, and the tension of strips is fine-tuned to be kept with slight fluctuation around 0 N by handle control system of MTS, observed with the test software. The upper fixture moved upward at a constant speed. Based on previous literature, the viscosity effect of tissue in tensile process can be minimized with speed of 6 mm/min, and the difficulty of strain rate control in tensile process can also be minimized [10]. Tensile load and displacement data were recorded at an interval of 0.01 s. (1) After the tissue strips were loaded and installed, each sample is implemented with 10 cycles of stretch-relaxation preloading at the rate of 0.08 N/s and a force range of 0-0.08 N; (2) the strain relaxation test is carried out on the samples after predebugging tensile treatment. After stretching the sclera sample with the rate of 6 mm/min for 0.1 mm, the displacement is terminated and maintained for 600 seconds. After 300 seconds of completely unloading of the external force, the sclera strips were performed with tensile fracture test, stretching the sclera until it is broken. In order to avoid scleral tissue dry in the experimental process, physiological saline sprayer is required to keep tissues moist. Throughout the experiment, a force-displacement curve is automatically obtained by the computer, and time, force, displacement, and other related data were automatically recorded. Relevant biomechanical parameters were calculated through the Excel table in the later stage, such as strain, instantaneous relaxation modulus, equilibrium relaxation modulus, elastic modulus *E*, and shear modulus *G*.

### 2.4. Creep Test

To obtain another viscoelastic of the scleral strips, creep properties should be tested. In this experiment, the upper and lower fixtures of MTS held the extension clips of scleral strips, and the tension of strips is fine-tuned to be kept with slight fluctuation around 0 N by handle control system of MTS. (1) After the tissue strips were loaded and installed, each sample is implemented with 10 cycles of stretch-relaxation preloading at the rate of 0.08 N/s and a force range of 0-0.08 N; (2) The creep test is carried out on the samples after predebugging tensile treatment. After stretching the sclera sample to 1.5 N with rate of 6 mm/min, the tension is controlled invariant and maintained for 600 s. Force-displacement curve is automatically obtained on the computer, and time, force, displacement, and other related data were automatically recorded. In order to avoid dry scleral tissue in the stretching process, physiological saline sprayer is required to keep tissues moist in entire period. Creep-related parameters, such as initial creep rate and equilibrium rate, were calculated through the Excel table in the later stage.

### 2.5. Bionanoindentation Test

In order to obtain the compression modulus of LC and inner surface of sclera, bionanoindentor (PIUMA, Optics 11, Netherlands) is used as the experimental tool. The ONH tissues were trimmed along the edge carefully under the microscope, and residual tissues on the surface of ONH were removed to expose the LC tissue. The optic nerves in posterior eyeball were cut to length of 5 mm. The ONH and ppSCl (remaining from previous experiments) were placed in petri dish with inner surface faced upward and marked each quadrant. A small amount of HBS solution is put into the petri dish, just coving the surface of tissue. Zero-load calibration is carried out after connecting indentation probe with indentation instrument. The indentation probe is moved downwards to 1 cm above the LC by manual adjustment, and then move the probe directly above the position to be measured. Probe is moved downwards by Optics 11 control operation system and compressed tissue slowly. The mean value is taken by 5 times of measurement per quadrant. Optics 11 data processing software is used to analyze the compressive modulus values of each part of the tissue.

### 2.6. Statistical Method

The data obtained were analyzed with SPSS 19.0 (SPSS for Windows 22.0) statistical analysis software and Excel 2016 database. The values of each group were expressed as mean ± standard deviation ($\bar{x}\pm s$). Paired-samples *T* test is applied in the intergroup comparison in each quadrant; ANOVA method and post hoc test (LSD, Tukey HSD) were applied in the intragroup comparison in each group. When *P* < 0.05, the difference is considered as statistically significant.

## 3. Results

### 3.1. Establishment of Elevated IOP Model in Tibetan Macaques

The elevated IOP models were constructed by injecting 0.2 ml *α*-chymotrypsin into the right anterior chamber of 8 Tibetan macaques. The IOP is stable at about 35 mmHg. The IOP in the experimental group is significantly increased compared with the control group, with significant difference (*P* < 0.01) (Table 1).

### 3.2. Changes of External Diameter of the Eyeball and ppSCl Thickness

**Figure 3:**
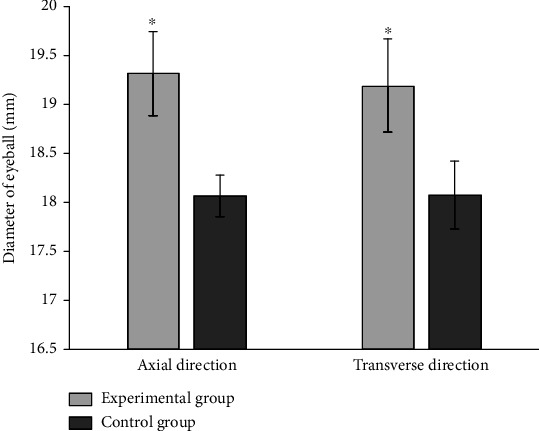
Axial and transverse diameters of the eyeballs in the experimental group and control group (mm).

The mean axial diameters and mean equatorial transverse diameters of the eyeballs were 18.07 ± 0.18 mm and 18.08 ± 0.32 mm in the control group, and 19.32 ± 0.39 mm and 19.19 ± 0.44 mm in the experimental group. The differences of these two parameters between the experimental group and the control group were statistically significant (*P* < 0.01). The diameters of the eyeballs in axial direction were equivalent to the equatorial transverse diameter in either the experimental group or the control group (*P* > 0.05) (Table 2). The ratio of axial diameter change between experimental group and control group is larger than transverse diameter change (Figure 3).

**Figure 4:**
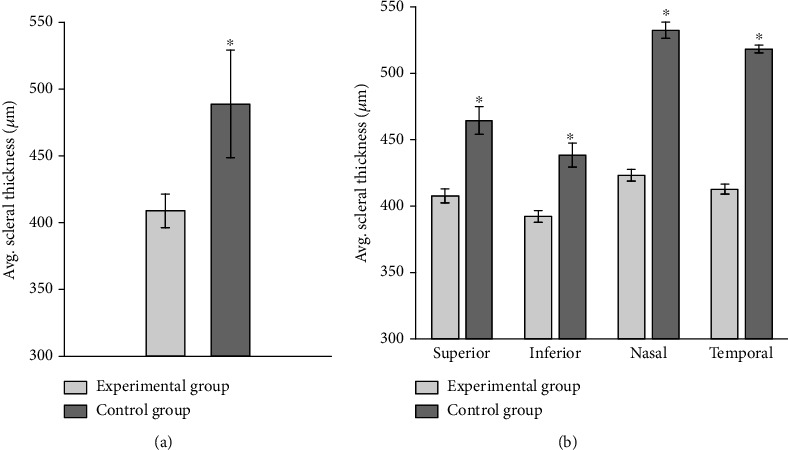
(a) Average thickness of peripapillary sclera in the experimental group and control group (*μ*m). (b) Average thickness of peripapillary sclera in each quadrant of experimental group and control group (*μ*m).

Under the microscope, the thickness of the ppSCl is measured with a microscopic scale. The average scleral thickness in the experimental group is 408.88 ± 11.82 *μ*m, thinner than that in the control group, 488.38 ± 38.96 *μ*m (Figure 4(a)). The scleral thickness of each quadrant is thinner in the experimental group than that in the control group, with significant difference (*P* < 0.01). Scleral thickness is different between four quadrants both in experiment and control group, with statistically significant (*P* < 0.01) (Figure 4(b), Table 3). The nasal sclera is the thickest and the inferior sclera is the thinnest both in experiment and control group.

### 3.3. Scleral Stress Relaxation Test

**Figure 5:**
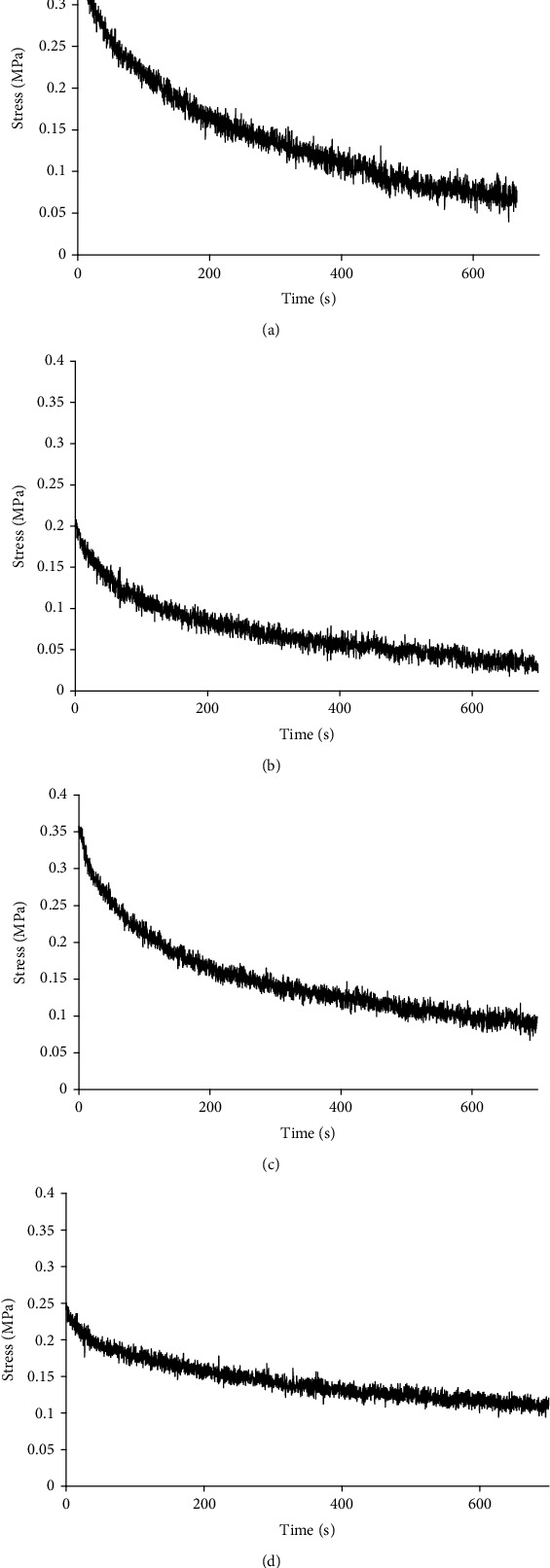
Result of peripapillary scleral stress relaxation test. (a) (Experimental group): stress relaxation curve of inferior sclera in axial direction. (b) (Control group): stress relaxation curve of inferior sclera in axial direction. (c) (Experimental group): stress relaxation curve of nasal sclera in circumferential direction. (d) (Control group): stress relaxation curve of nasal sclera in circumferential direction.

After constant displacement is controlled with 600 s, the original data recorded by the computer were input into the Excel table. With time as the abscissa and stress as the ordinate, the stress relaxation curve of scleral strip is obtained with graphing procedure. Displacement, width, and length of tissue were used to calculate strain (*μ*m/min). The instantaneous relaxation modulus is calculated with the stress and strain at the initial stage of stress (*t*_0_). As the curve reached a steady state (*t*_∞_), the equilibrium relaxation modulus of sclera is calculated with stress and strain. Scleral tissues show stress relaxation characteristics both in the experimental group and control group. In the experimental group, the instantaneous relaxation modulus and equilibrium relaxation modulus of scleral strip were higher than those in the control group (Figures 5(a)–5(d)). Whether ppSCl in axial direction or in circumferential direction, statistical analysis indicates that the differences between two groups were remarkable in each quadrant (*P* < 0.05). The results suggest that under the same displacement condition, scleral tissues with normal IOP had better extended adaptability and tissue viscoelastic behavior. Moreover, scleral tissues in the control group had preferable extended reserve for the passive tension produced by external forces (Tables 4 and 5). The relaxation modulus of temporal scleral tissue is fairly high in circumferential direction (*P* < 0.05), suggesting that the temporal ppSCl may have poor viscoelastic behavior. The result also showed that elevated IOP has a greater effect on the instantaneous relaxation modulus in circumferential direction as well as equilibrium relaxation modulus in axial direction.

### 3.4. Creep Test of Scleral Tissue

After constant force is controlled with 600 s, the original data recorded by the computer were input into the Excel table. With time as the abscissa and strain as the ordinate, the creep curve of scleral strips is obtained with graphing procedure. Displacement, width, and length of tissue were used to calculate strain (*μ*m/min). The first-stage data of creep curve were selected to calculate the initial creep rate, and the second-stage data of creep curve were selected to calculate the equilibrium creep rate.

**Figure 6:**
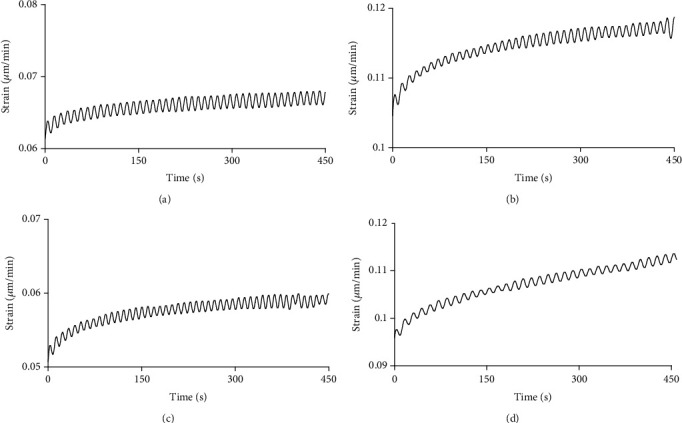
Result of peripapillary scleral creep test. (a) (Experimental group): creep curve of inferior sclera in axial direction. (b) (Control group): creep curve of inferior sclera in axial direction. (c) (Experimental group): creep curve of nasal sclera in circumferential direction. (d) (Control group): creep curve of nasal sclera in circumferential direction.

The scleral tissues presented creep characteristics both in the experimental group and the control group. In the experimental group, the initial and equilibrium creep rates of scleral strips were slower than those in the control group (Figures 6(a)–6(d)). In each quadrant, the initial rate and equilibrium rate of each quadrant were lower in the experimental group than that in the control group, with significant difference (*P* < 0.05). The initial rate has significant difference between each quadrant in double groups, and equilibrium rate has significant difference between each quadrant in the experimental group (*P* < 0.05). The equilibrium rate between each quadrant has no significant difference in the control group (*P* > 0.05) (Tables 6 and 7). These results suggest that scleral tissues of the control group may have better creep performance and better adaptability to external forces. In the control group, the initial and equilibrium creep rates of ppSCl strips were different between axial direction and circumferential direction, suggesting that sclera is of anisotropic characteristic.

### 3.5. Scleral Tissue Tensile Test

**Figure 7:**
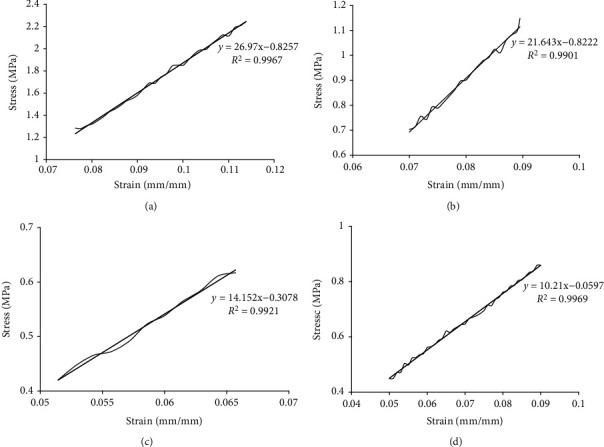
Curve and trendline of peripapillary scleral stress-strain test. (a) (Experimental group): temporal sclera in circumferential direction. (b) (Experimental group): superior sclera in circumferential direction. (c) (Control group): temporal sclera in circumferential direction. (d) (Control group): superior sclera in circumferential direction.

Tensile tests of scleral strips showed that the stress-strain relationship of scleral tissues in the early stage of stretching presented positive proportional relationships, which complies with Hooke's Law. The stress and strain at this stage presented as linearly change, and the strips were in the stage of elastic deformation. The original data recorded by MTS is input into the Excel table. Displacement, width, and length of tissue were used to calculate strain (*μ*m/min). With strain in the selected section as the abscissa and stress as the ordinate, the stress-strain curve is with graphing procedure. The function relation and elastic modulus E were obtained with calculation. Under the same tensile conditions, the same strain of ppSCl tissue in the experimental group requires more stress compared to the control group. The steeper ascent curves of stress indicated that the tissues were stiffer and of worse organizational compliance to external forces (Figures 7(a)–7(d)).

In the experimental group, the mean elastic modulus (*E*) of the ppSCl tissue in circumferential direction is 23.28 ± 2.19 MPa, more than that in the control group (*P* < 0.05). The largest *E* value is in the temporal quadrant and the smallest in the nasal quadrant (Table 8). The mean *E* value of ppSCl tissue in axial direction is 22.15 ± 2.22 MPa, more than that in the control group (*P* < 0.05). The largest *E* value is in the superior quadrant and the smallest in nasal quadrant (Table 9). In the control group, the mean *E* of ppSCl tissue is 10.98 ± 1.74 MPa in circumferential direction and 9.55 ± 1.62 MPa in axial direction. The *E* value of temporal quadrant is larger than that of the other quadrants both in two direction (*P* < 0.05) (Tables 8 and 9). In the experimental group, the difference of *E* value between four quadrants in the circumferential direction had not statistically significant (*P* > 0.05), but had statistically significant in axial direction (*P* < 0.05). The *E* value of each quadrant in the experimental group is approximately 2.5 times than that in the control group.

Based on the elastic range, the relationship between shear modulus *G* and elastic modulus *E* is shown as this formula, *G* = *E*/(2(1 + *μ*)). According to the literature report, Poisson's ratio (*μ*) is set to be 0.46, so the shear modulus of scleral tissue can be obtained (Table 10).

### 3.6. Bionanoindentation Test in LC and ppSCl

**Figure 8:**
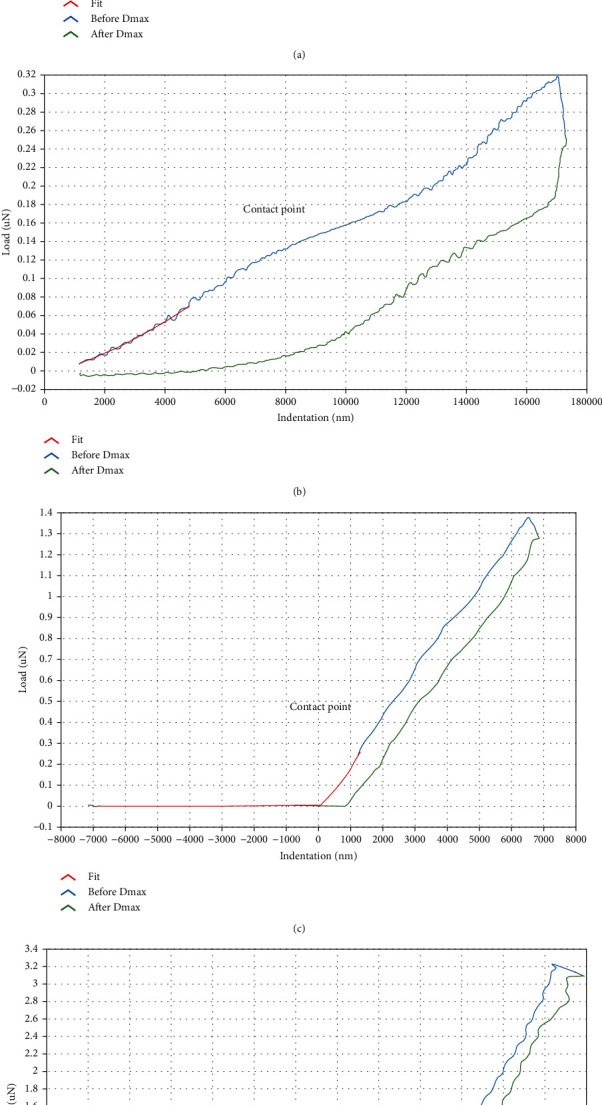
Compression force-displacement (CFD) curve of nanoindentation test. (a) CFD curve of LC in the control group (nasal side). (b) CFD curve of LC in the experimental group (temporal side). (c) CFD curve of peripapillary sclera in the control group (temporal side). (d) CFD curve of peripapillary sclera in the experimental group (nasal side).

The measurement is proceeded automatically with matching software of PIUMA. After the force-displacement curve is obtained, fitting calculation is made to obtain the value of compressive modulus based on the curvature of the curve, the size of probe, and depth of compression, etc. (Figure 8). When downward pressure is loaded and unloaded to the LC and ppSCl surface, the force-displacement curve under loading did not coincide with the curve under unloading, and the LC and ppSCl presented with elastic hysteresis. In the experimental group, LC is excessively soft, the rebound delay phenomena of indentation probe in unloading process is obvious than that in the control group, and the curve had considerable fluctuation in the process of force loading and unloading. The hardness of ppSCl tissue is larger than LC, and the curves in force loading and unloading process were closer than LC (Figure 8).

**Figure 9:**
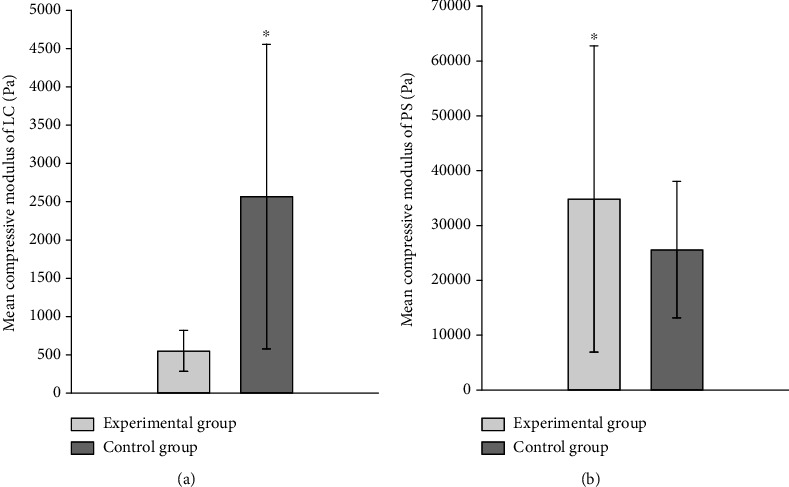
Comparisons of mean compressive modulus (Pa) of LC (a) and peripapillary sclera (b) between experimental and control group.

The compressive modulus of LC were smaller in the experimental group than control group, and the differences were statistically significant (*P* < 0.05). The compressive modulus of ppSCl were higher in the experimental group than control group (*P* < 0.05) (Figure 9, Table 11).

## 4. Discussion

The sclera is the hardest tissue in the eye and plays an important role in maintaining the shape of the eye. It consists of the surface sclera, scleral parenchyma, and brown-black layer. Scleral parenchyma contains dense collagen fiber bundles that are arranged in parallel on the outer surface and interlaced on the inner surface. The brown-black layer contains smaller collagen fibers bundles. Collagen fibers play an important role in maintaining the structure, function, and biomechanical properties of sclera [11]. In posterior sclera, two-thirds of outer part forms the sheath of optic nerve, and the inner part forms LC [12, 13]. Some studies showed that the sclera had the characteristics of nonlinearity, anisotropy, and viscoelasticity [14].

### 4.1. The Impact of Elevated IOP on the Structure of the Eyeball

Previous investigations have revealed that elevated IOP increases the axis length of human eyes. Researchers believed that the mechanism of increased axial length caused by the rise in IOP may be related to the elongated scleral fibers [15]. But other researchers found that the axis length in some patients can be partially restored accompanied by IOP reduced with timely and effective treatment [16, 17]. As a container, interior of the eyeball expands with elevated IOP based on stretching and elongation of the scleral fibers, and scleral fibers extend in all directions due to anisotropic characteristics. However, it remains unclear whether the increased external diameter of the eyeball is mainly in axial or transverse direction with elevated IOP. In this study, we find that the axial diameter of the eyeballs are almost equivalent to that of the transverse diameter in elevated and normal IOP models. But the axial and transverse diameters of the eyeballs with elevated IOP are longer than that with normal IOP. In addition, the difference between axial and transverse diameters with elevated IOP is partial significant than that with normal IOP. Scleral extension and resistance are mainly depending on scleral fibrous tissue, and scleral fibrous tissue has anisotropic arrangement. Therefore, it can be inferred that elevated IOP may have an impact on the dominant mechanical direction of scleral fibers.

Few studies have been done on the impact of elevated IOP on scleral thickness of posterior eyeball, because of the limitation on the accurate detection methods for posterior scleral thickness measurement. Some investigators thought that the posterior sclera was thickened with elevated IOP [18]. In this study, we find that nasal sclerae are the thickest and inferior sclerae are the thinnest both in elevated and normal IOP models. The thickness of posterior sclera around ONH with elevated IOP is thinner than that with normal IOP in four quadrants (*P* < 0.01). These results are consistent with one previous report [19]. The possible reason for the thinning of sclera with elevated IOP is that when sclera are passive stretched, the original curling state of scleral fibers is changed into a stretching state. It is difficult to recover to normal state with long-term effects of elevated IOOP, leading to thinning of the sclera.

### 4.2. Impact of Elevated IOP on the Mechanical Properties of Scleral Tissues

In this study, we found that the instantaneous and equilibrium relaxation modulus of sclera with normal IOP between four quadrants have significant difference both in circumferential and axial direction (*P* < 0.05), indicating that sclera with normal IOP has anisotropic features. The instantaneous relaxation modulus of sclera in four quadrants with elevated IOP are greater than that with the normal group both in the axial direction and the circumferential direction around ONH (*P* < 0.05), and the instantaneous and equilibrium relaxation modulus of sclera with elevated IOP between four quadrants have no significant difference in axial direction (*P* > 0.05). Data from the creep tests suggest that under external forces, sclera is difficult to extend further through the elastic action of tissue fibers, and the compliance is decreased. These results indicate that the elevated IOP may have exact impact on elastic mechanical and anisotropic characteristics of sclera. The stress relaxation ability of sclera is weakened and the hardness of the tissues is increased. We can infer that when the chronic glaucoma patients are exposed to moderate ocular hypertension for a long time, ppSCl is difficult to effectively resist the further passive extension caused by elevated IOP, and the tissues were difficult to return to initial state even if the IOP is reduced.

According to research finding, the biomechanical properties of ppSCl are changed with elevated IOP, and these changes can be transmitted to the ONH, which contributes to the characteristic changes of ONH structure in glaucoma [20]. The results of tensile tests in this research show that the elastic modulus of sclera with elevated IOP is approximately 2 times higher than that with the normal group in four quadrants (*P* < 0.01). These data suggest that with long-term exposure to elevated IOP, scleral tissue hardness is increased while the elasticity is decreased. The external forces required for scleral tissue deformation would be greater. In the control group, the elastic modulus of ppSCl in circumferential is higher than that in axial direction, which suggests that the resistance of circumferential sclera is higher than that of axial sclera under passive stretch caused by elevated IOP. Hence, it can be inferred that the alignment of scleral fibrous tissue is dominant in the circumferential direction around the ONH. The elastic modulus of ppSCl was higher with elevated IOP than that with normal IOP both in circumferential and axial directions, and elastic features of sclera between four quadrants become less pronounced with elevated IOP, which suggest that the elastic and anisotropic characteristics of scleral fibrous tissues can be changed due to long-term effects of external forces.

For some familiar materials, there is a positive proportional relationship between the thickness and stiffness. The thicker the material, the stronger it resists to deformation. In this study, the posterior scleral is the thickest in nasal quadrant with normal IOP, but the elastic modulus and shear modulus of the nasal sclera were not the largest. The possible reason for this phenomenon would be that at low IOP, scleral fibrous tissue is slightly bent, and the compliance is strong at this point. When IOP increased, the scleral fibers are stretched straight, which leads an increase in the stiffness of the scleral fibers. This indicated that the nasal sclera has a greater compliance reserve. With ocular hypertension, denaturation of tissues due to tensile action of fibers should not be ignored. Predictably, it is incorrect to simply believe that with the existence of special structures like scleral fibers, there is a positive relationship between the rigidity and thickness.

Some studies have shown that changes in scleral thickness and viscoelasticity caused by long-term effects of elevated IOP are relative to the remodeling of extracellular matrix (ECM) [21, 22]. It was found that under abnormal strain loading condition, fibroblasts secrete related factors, causing changes in the ECM around cells [23]. Force-induced changes of expression of the ECM proteins and associated factors may increase the resistance to further external forces, preventing further damage to tissues and cells. Nanoindentation tests show that compressive modulus of ppSCl has a marked increase with elevated IOP, suggesting that the scleral tissues may undergo fibrosis. However, few studies have addressed the cause of scleral hardening, and the mechanisms underlying the ECM remodeling remain to be determined. In high myopia eyes, the biomechanical properties and thickness of sclera were different from normal eyes, and the ECM remodeling was found [24, 25]. Similarly, we speculate that sclera with elevated IOP may have similar pathophysiological processes of the sclera with high myopia.

### 4.3. Effect of Elevated IOP on Biomechanical Characteristics of LC

In this study, compressive modulus of LC in nasal quadrant is greater than that in other three quadrants with normal IOP. We also find that the compressive modulus decreases gradually from the middle to the periphery in the course of research, which may have a connection with its structural characteristics. Compression modulus of each quadrant at the LC is higher with elevated IOP than that with normal IOP. These results indicate that with elevated IOP, the LC is softened and the effect of resisting external forces is reduced. Previous studies have shown that in the model of human and monkey eye with glaucoma, the ECM of LC changed [26–28]. The density of collagen fibers of LC decreased significantly in human with elevated IOP [29], possibly due to degeneration of local tissues or alteration of blood flow [30]. These findings may interpret results in our research. The LC has smaller shape and deeper position, and there has been no effective methods for direct measurement of its biomechanical characteristics. Previous researches on biomechanics of LC were mainly based on MRI or OCT. By detecting the displacement changes under different IOP, biomechanical characteristics were analyzed by simulation with finite element model (FEM) [31, 32]. In this study, we used a bionanoindenter to measure biomechanical characteristics of LC. Compared with previous studies on LC, the actual compression modulus is less than the simulation value. The simulation data from FEM showed that the approximate compressive modulus of the LC is 50 kPa [32]. The average measured value in our research is 5.60 ± 0.13 kPa with normal IOP and 0.47 ± 0.13 kPa with elevated IOP. It suggests that more authentic biomechanical value of the LC can be obtained through nanoindentation test. These results provide a theoretical basis for the further explanation of the glaucomatous optic nerve damage.

Some studies suggest that under long-term effects of ocular hypertension, the increase in scleral hardness was a protective mechanism, which resisted external forces transmitting to the ONH. But clinical evidences proved that this “protective mechanism” was unable to protect the optic nerve effectively, but is rather a passive degeneration of scleral tissue with elevated IOP. Studies have shown that IOP-related stress keeps constant between eyeballs with same geometric shape, but under the same mechanical load, the differences in local morphological changes within the sclera hinges on material property of sclera. If material property of sclera around ONH changed, the local deformation of the eyeball will be different under the same level of IOP. Therefore, ppSCll deformation induced by elevated IOP would be a key factor to the biomechanical abnormality of ONH [33]. Reduced scleral elasticity leads to more IOP conduction to ONH, causing the damages of LC structure. Meanwhile, the blood supply from branches of posterior ciliary artery may also be reduced due to the scleral deformation and leads insufficient blood supply for the LC and changes in glaucomatous optic nerve.

## 5. Conclusions

Based on this study, we can conclude that sclera has viscoelastic and anisotropic characteristics, and various biomechanical characteristics of sclera and LC can be affected with elevated IOP. We can infer from results that with IOP rises suddenly, sclera is able to have accompanied elongation, and the pressure is equally distributed to all parts of ppSCl, which has no serious impact on ONH. Once the IOP decreased promptly, the biomechanical characteristics of sclera may return to initial state gradually. With the elevated IOP for a long time, sclera can be more stiffer and less elastic due to scleral degeneration and/or changes in internal structure. Its resistance reserve to further external forces reduces so that the sclera cannot relieve the IOP effectively by extension of scleral fibers. At this stage, IOP may be distributed more to the ONH gradually, making the LC displaced backward. Accompanied with local degeneration and insufficient blood supply, the LC tissue is softened and unable to resist the effect of elevated IOP, resulting in further damages of LC and optic nerve. Finding ways to restore the biomechanical characteristics of the sclera and LC with elevated IOP is promising to prevent the progression of glaucoma.

## Figures and Tables

**Table 1:** Average IOP (mmHg) in the experimental group (Exp) and the control group (Ctrl).

Groups	1	2	3	4	5	6	7	8	$\bar{x}\pm s$
Exp.	36.86	33.59	33.42	36.15	35.96	34.33	33.18	32.96	34.56 ± 1.54^∗∗^
Ctrl.	20.11	19.25	19.76	20.25	18.49	19.36	20.51	17.18	19.36 ± 1.09

IOP of each eye was measured for 3 times repeatedly. The IOP in the experimental group was significantly increased compared with the control group, with significant difference (*P* < 0.01).

**Table 2:** Axial diameter and transverse diameter of the eyeballs in experimental and control group (mm, $\bar{x}\pm s$).

Groups	Axial diameter (avg.)	Transverse diameter (avg.)	*P*
Exp.	19.32 ± 0.43	19.19 ± 0.47	0.31
Ctrl.	18.07 ± 0.19	18.08 ± 0.34	0.79
*P*	0.001^∗∗^	0.002^∗∗^	

Axial and transverse diameter of the eyeballs in each group was measured for 3 times repeatedly. The axial and transverse diameters of the eyeballs were longer in the experimental group than those in the control group, with significant difference (*P* < 0.01).

**Table 3:** Peripapillary scleral thickness in each quadrant of experimental group and control group (*μ*m).

Groups	Scleral thickness (*μ*m, $\bar{x}\pm s$)				*F*	*P*
	Superior	Inferior	Nasal	Temporal		
Exp.	407.75 ± 4.32	392.25 ± 3.49	423.25 ± 3.49	412.75 ± 2.86	38.97	≤0.001^∗∗^
Ctrl.	464.50 ± 9.53	438.50 ± 8.20	532.50 ± 5.17	518.25 ± 2.17	124.52	≤0.001^∗∗^
*P*	0.006^∗∗^	0.001^∗∗^	≤0.001^∗∗^	≤0.001^∗∗^		

Thickness of each scleral trip in each group was measured for 3 times repeatedly. Paired-samples test was applied in the intergroup comparison in each quadrant; ANOVA method and post hoc test (LSD, Tukey HSD) were applied in the intragroup comparison in each group. The scleral thickness of each quadrant was thinner in the experimental group than that in the control group, with significant difference (*P* < 0.01). Scleral thickness of each group was significantly different in each quadrant, respectively (*P* < 0.01).

**Table 4:** Instantaneous modulus and equilibrium modulus of stress relaxation of ppSCl in circumferential direction (MPa, Inst: instantaneous modulus; Equl: equilibrium modulus).

Groups	Modulus of scleral stress relaxation (MPa)				$\bar{x}\pm s$	*F*	*P*
	Superior	Inferior	Nasal	Temporal			
Exp. (Inst.)	21.51 ± 1.42	23.36 ± 0.79	19.97 ± 0.19	26.23 ± .077	22.76 ± 2.49	8.80	0.031^∗^
Ctrl. (Inst.)	15.42 ± 1.61	12.19 ± 0.94	14.12 ± 0.36	18.85 ± 0.27	15.14 ± 2.61	8.55	0.033^∗^
*P*	0.019^∗^	0.012^∗^	0.005^∗^	0.013^∗^			
Exp. (Equl.)	5.98 ± 0.41	13.07 ± 0.85	5.08 ± 0.05	14.74 ± 0.77	9.72 ± 4.28	64.90	0.001^∗^
Ctrl. (Equl.)	3.49 ± 0.32	2.07 ± 0.11	2.64 ± 0.12	6.61 ± .026	3.71 ± 1.76	13.33	0.015^∗^
*P*	0.040^∗^	0.006^∗^	0.003^∗^	0.009^∗^			

Modulus of scleral stress relaxation of each scleral trip was measured for 3 times repeatedly. Paired-samples test was applied in the intergroup comparison in each quadrant; ANOVA method and post hoc test (LSD, Tukey HSD) were applied in the intragroup comparison in each group. The instantaneous relaxation modulus and equilibrium relaxation modulus of each quadrant were higher in the experimental group than that in the control group, with significant difference (*P* < 0.05). The instantaneous relaxation modulus and equilibrium relaxation modulus of each group were significantly different in each quadrant, respectively (*P* < 0.05).

**Table 5:** Instantaneous modulus and equilibrium modulus of stress relaxation of ppSCl in axial direction (MPa, Inst: instantaneous modulus; Equl: equilibrium modulus).

Groups	Modulus of scleral stress relaxation (MPa)				$\bar{x}\pm s$	*F*	*P*
	Superior	Inferior	Nasal	Temporal			
Exp. (Inst.)	24.02 ± 0.45	22.65 ± 1.24	21.23 ± 0.85	21.88 ± 1.65	22.44 ± 1.64	1.11	0.442^#^
Ctrl. (Inst.)	13.68 ± 0.09	12.34 ± 0.59	11.26 ± 0.89	14.03 ± 1.22	12.82 ± 1.46	2.46	0.202^#^
*P*	0.002	0.017	0.015	0.034			
Exp. (Equl.)	5.45 ± 1.09	6.48 ± 0.71	4.33 ± 0.06	7.66 ± 2.51	5.98 ± 2.01	1.02	0.472^#^
Ctrl. (Equl.)	2.81 ± 0.02	2.07 ± 0.09	2.12 ± 0.04	4.66 ± 0.64	2.91 ± 1.17	14.02	0.013^∗^
*P*	0.045	0.025	0.001	0.048			

Modulus of scleral stress relaxation of each scleral trip was measured for 3 times repeatedly. Paired-samples test was applied in the intergroup comparison in each quadrant; ANOVA method and post hoc test (LSD, Tukey HSD) were applied in the intragroup comparison in each group ^#^Instantaneous relaxation modulus of each quadrant in each group, equilibrium relaxation modulus of each quadrant in the experimental group had no significantly different (*P* < 0.05). Equilibrium relaxation modulus of each quadrant had significant difference in the control group (*P* < 0.05). The instantaneous and equilibrium relaxation modulus of each quadrant were higher in the experimental group than that in the control group, with significant difference (*P* < 0.05)

**Table 6:** Initial rate and equilibrium rate of creep of ppSCl in circumferential direction (*μ*m/min, Int: initial rate; Equl: equilibrium rate).

Groups	Rate of creep (*μ*m/min)				$\bar{x}\pm s$	*F*	*P*
	Superior	Inferior	Nasal	Temporal			
Exp. (Int.)	4.65 ± 0.17	3.01 ± 0.06	3.12 ± 0.18	4.05 ± 0.11	3.73 ± 0.69	54.59	0.001^∗^
Ctrl. (Int.)	6.02 ± 0.13	5.66 ± 0.35	4.35 ± 0.15	5.46 ± 0.24	5.34 ± 0.63	10.53	0.023^∗^
*P*	0.021^∗^	0.018^∗^	0.025^∗^	0.017^∗^			
Exp. (Equl.)	0.61 ± 0.08	0.28 ± 0.04	0.24 ± 0.03	0.54 ± 0.05	0.41 ± 0.16	55.43	0.001^∗^
Ctrl. (Equl.)	1.25 ± 0.06	1.23 ± 0.11	1.35 ± 0.16	1.42 ± 0.21	1.33 ± 0.23	5.56	0.066^#^
*P*	0.006^∗^	0.013^∗^	0.016^∗^	0.045^∗^			

Creep rate of each scleral trip was measured for 3 times repeatedly. Paired-samples test was applied in the intergroup comparison in each quadrant; ANOVA method and post hoc test (LSD, Tukey HSD) were applied in the intragroup comparison in each group. The initial rate and equilibrium rate of each quadrant were lower in the experimental group than that in the control group, with significant difference (*P* < 0.05). The initial rate had significant difference between each quadrant in double groups, and equilibrium rate had significant difference between each quadrant in the experimental group (*P* < 0.05). ^#^The equilibrium rate between each quadrant had no significant difference in the control group (*P* > 0.05).

**Table 7:** Initial rate and equilibrium rate of creep of ppSCl in axial direction (*μ*m/min, Int: initial rate; Equl: equilibrium rate).

Groups	Creep rate (*μ*m/min)				$\overset{¯}{x}\pm s$	*F*	*P*
	Superior	Inferior	Nasal	Temporal			
Exp. (Int.)	4.55 ± 0.05	4.15 ± 0.15	3.41 ± 0.12	4.16 ± 0.23	4.06 ± 0.46	16.04	0.011^∗^
Ctrl. (Int.)	6.23 ± 0.08	6.33 ± 0.16	4.85 ± 0.35	5.06 ± 0.25	5.63 ± 0.77	11.73	0.019^∗^
*P*	0.037^∗^	0.009^∗^	0.047^∗^	0.048^∗^			
Exp. (Equl.)	0.71 ± 0.06	0.37 ± 0.02	0.31 ± 0.01	0.52 ± 0.06	0.47 ± 0.17	19.62	0.007^∗^
Ctrl. (Equl.)	1.28 ± 0.03	1.17 ± 0.05	1.33 ± 0.02	1.35 ± 0.05	1.28 ± 0.06	7.66	0.039^∗^
*P*	0.011^∗^	0.001^∗^	0.003^∗^	0.006^∗^			

Creep rate of each scleral trip was measured for 3 times repeatedly. Paired-samples test was applied in the intergroup comparison in each quadrant; ANOVA method and post hoc test (LSD, Tukey HSD) were applied in the intragroup comparison in each group. The initial rate and equilibrium rate of each quadrant were lower in the experimental group than that in the control group, with significant difference (*P* < 0.05). The initial rate and equilibrium rate of each group were significantly different in each quadrant, respectively (*P* < 0.05).

**Table 8:** Elastic modulus *E* (MPa) of ppSCl in circumferential direction.

Groups	Elastic modulus *E* (MPa)				$\bar{x}\pm s$	*F*	*P*
	Superior	Inferior	Nasal	Temporal			
Exp.	22.75 ± 1.57	23.57 ± 0.95	20.75 ± 0.98	26.06 ± 1.29	23.28 ± 2.19	6.44	0.052^#^
Ctrl.	11.15 ± 0.27	9.02 ± 0.17	10.52 ± 0.44	13.21 ± 1.34	10.98 ± 1.74	11.53	0.019^∗^
*P*	0.009^∗^	0.002^∗^	0.047^∗^	0.011^∗^			

Paired-samples test was applied in the intergroup comparison in each quadrant; ANOVA method and post hoc test (LSD, Tukey HSD) were applied in the intragroup comparison in each group. The elastic modulus of each quadrant was higher in the experimental group than that in the control group, with significant difference (*P* < 0.05). The elastic modulus was different between each quadrant in the control group (*P* < 0.05). ^#^The elastic modulus had no difference between each quadrant in the experimental group (*P* > 0.05).

**Table 9:** Elastic modulus *E* (MPa) of ppSCl in axial direction.

Groups	Elastic modulus *E* (MPa)				$\bar{x}\pm s$	*F*	*P*
	Superior	Inferior	Nasal	Temporal			
Exp.	24.98 ± 0.08	22.57 ± 0.29	19.18 ± 0.37	21.88 ± 0.21	22.15 ± 2.22	169.92	≤0.001^∗^
Ctrl.	9.64 ± 0.22	9.72 ± 0.43	7.30 ± 0.23	11.54 ± 0.15	9.55 ± 1.62	78.53	0.001^∗^
*P*	≤0.001^∗^	0.001^∗^	0.002^∗^	≤0.001^∗^			

Paired-samples test was applied in the intergroup comparison in each quadrant; ANOVA method and post hoc test (LSD, Tukey HSD) were applied in the intragroup comparison in each group. The elastic modulus of each quadrant was higher in the experimental group than that in the control group, with significant difference (*P* < 0.01). The elastic modulus was different between each quadrant in each group (*P* < 0.01).

**Table 10:** Shear modulus *G* (MPa) of ppSCl (Circ: circumferential direction; Axl: axial direction).

Groups	Shear modulus *G* (MPa)				$\bar{x}\pm s$	*F*	*P*
	Superior	Inferior	Nasal	Temporal			
Exp. (Circ.)	7.79 ± 0.54	8.07 ± 0.33	7.11 ± 0.42	8.93 ± 0.44	7.97 ± 0.71	6.44	0.052^#^
Ctrl. (Circ.)	3.82 ± 0.09	3.09 ± 0.06	3.61 ± 0.15	4.52 ± 0.46	3.76 ± 0.54	11.53	0.019^∗^
*P*	0.001^∗^	0.002^∗^	0.006^∗^	0.010^∗^			
Exp. (Axl.)	8.55 ± 0.03	7.73 ± 0.12	6.57±	8.93 ± 0.25	7.59 ± 0.71	169.92	≤0.001^∗^
Ctrl. (Axl.)	3.31 ± 0.08	3.33 ± 0.15	2.51 ± 0.09	4.61 ± 0.04	3.27 ± 0.52	78.53	0.001^∗^
*P*	≤0.001^∗^	0.001^∗^	0.001^∗^	≤0.001^∗^			

Paired-samples test was applied in the intergroup comparison in each quadrant; ANOVA method and post hoc test (LSD, Tukey HSD) were applied in the intragroup comparison in each group. In circumferential and axial direction, the shear modulus of each quadrant was higher in the experimental group than that in the control group, with significant difference (*P* < 0.01). In axial direction of each group and circumferential direction of control group, shear modulus was different between each quadrant (*P* < 0.01). ^#^In circumferential direction of experimental group, shear modulus had no different between each quadrant (*P* > 0.05).

**Table 11:** Mean value of compressive modulus in each quadrant of LC and ppSCl (Pa).

Groups	Compression modulus (Pa)				$\bar{x}\pm s$
	Superior	Inferior	Nasal	Temporal	
Exp. (LC)	265.08 ± 17.99	335.66 ± 34.16	952.81 ± 41.77	474.32 ± 128.62	547.58 ± 257.12
Ctrl. (LC)	3526.03 ± 344.39	5040.21 ± 2476.49	7487.27 ± 2785.26	5601.23 ± 125.06	2566.96 ± 1977.86
*P*	0.047^∗^	0.012^∗^	0.015^∗^	0.048^∗^	
Exp. (PS.)	23090.31 ± 8141.27	23663.2 ± 7731.49	97254.71 ± 28789.66	17836.86 ± 19933.37	34814.45 ± 27773.01
Ctrl. (PS.)	7778.80 ± 3609.59	6261.27 ± 3345.25	74823.64 ± 32135.76	30450.03 ± 10970.29	25596.99 ± 12257.23
*P*	0.023^∗^	0.035^∗^	0.048^∗^	0.044^∗^	

Paired-samples test was applied in the intergroup comparison in each quadrant. Compressive modulus of each quadrant of LC was lower in the experimental group than that in the control group, with significant difference (*P* < 0.05). Compressive modulus of each quadrant of ppSCl was higher in the experimental group than that in the control group, with significant difference (*P* < 0.05).

## Data Availability

The labeled dataset used to support the findings of this study are available from the corresponding author upon request.
